# Editorial: Utilizing real-world evidence for better endocrine health management

**DOI:** 10.3389/fendo.2026.1846678

**Published:** 2026-04-24

**Authors:** Meng Mei, Luciane Cruz Lopes, Cao Li

**Affiliations:** 1 Department of Pharmacy, Wuhan Children’s Hospital (Wuhan Maternal and Child Healthcare Hospital), Tongji Medical College, Huazhong University of Science and Technology, Wuhan, China; 2 Graduate Course in Pharmaceutical Sciences, University of Sorocaba, Sorocaba, Brazil; 3 Department of Pharmacy, Beijing Tiantan Hospital, Capital Medical University, Beijing, China

**Keywords:** clinical management, endocrine diseases, evidence-based medicine, real-world data, real-world evidence

Endocrine disorders span a diverse spectrum from common chronic conditions such as diabetes mellitus to rare inherited metabolic disorders and hormone-dependent reproductive issues, collectively imposing a heavy burden on global public health and often leading to premature morbidity, mortality and reduced quality of life in affected populations (1–4). While traditional randomized controlled trials (RCTs) represent the gold standard for evaluating the efficacy and safety of endocrine interventions, their stringent eligibility criteria, substantial costs, ethical constraints, and limited external validity give rise to critical evidence gaps in real-world clinical practice. Consequently, real-world data (RWD) and real-world evidence (RWE) derived from routine clinical care have emerged as essential complements to RCTs (5, 6). The 12 studies featured in this Research Topic vividly demonstrate the multi-dimensional value of RWD/RWE in the field of endocrinology, covering key clinical applications including endocrine disease risk prediction and stratification, accurate diagnosis and screening, treatment strategy optimization and post-marketing pharmacovigilance.

A core strength of RWD is to identify context-specific risk factors and develop clinically actionable prediction models for diverse endocrine populations. Li et al.‘s retrospective analysis of 28,580 hospitalized diabetes patients identified type 1 diabetes (T1DM), impaired renal function, and insulin or glinide use as key risk factors for hypoglycemia, with distinct temporal patterns, providing a basis for targeted inpatient monitoring and intervention strategies. For type 2 diabetes (T2DM) complications, Pan et al. validated the triglyceride-glucose index and triglyceride to high-density lipoprotein cholesterol ratio as accessible biomarkers for white matter hyperintensities, enabling early cerebrovascular risk stratification in routine clinical practice. In rare and specialized populations, RWD fills critical evidence voids that RCTs fail to address. Hua et al. developed and validated a nomogram for predicting dyslipidemia risk in 913 children with Wilson disease. This model integrated six clinical variables including age group, ALT, GGT, Hcy, SOD and PLT, and showed robust predictive performance, filling the gap in personalized assessment tools for rare pediatric endocrine diseases and facilitating early clinical intervention. Shen et al. elucidated the correlation between higher pre-operative white blood cell count to hemoglobin ratio with reduced postoperative Physical Component Scores in endometriosis patients, while Wu et al. demonstrated an independent negative association between serum apolipoprotein B-100 and lumbar bone mineral density in postmenopausal women, particularly in younger or obese subgroups. These studies fully highlight RWD’s capacity to refine risk stratification across the entire endocrine disease spectrum and guide clinical decision-making for different populations.

Traditional screening methods for endocrine diseases such as diabetes often suffer from high false-negative rates, leading to missed diagnoses and delayed clinical interventions (7, 8). Yi et al.‘s study addressed this clinical challenge by developing a diabetes screening model integrating chest CT pancreatic radiomics features with laboratory data. It effectively identifies undiagnosed diabetes patients with normal fasting glucose, overcoming a critical limitation of conventional fasting plasma glucose-based screening. This RWD-driven approach leverages existing clinical data to improve diagnostic accuracy without additional burdens on patients or healthcare systems.

RWD enables the assessment of therapeutic interventions in real-world diverse patient populations, supplementing and expanding on RCT results. Niu et al.‘s multi-institutional retrospective analysis compared the levonorgestrel-releasing intrauterine device with oral norethindrone in adenomyosis patients, identifying key clinical trade-offs. Compared with oral norethindrone, the levonorgestrel-releasing intrauterine device lowers pelvic inflammatory disease and severe anemia risk but raises bacterial vaginosis risk, offering actionable evidence to guide personalized treatment decisions. Avcı et al.‘s longitudinal and comparative study documented significant reductions in fat-free mass, fat-free mass index, and phase angle in 20 assigned male at birth individuals undergoing feminizing gender-affirming hormone therapy over 12 months. These findings underscore the critical role of RWD in optimizing clinical management for marginalized and understudied populations often excluded from RCTs, by offering unique insights into treatment-related physiological changes and supporting clinical management refinement.

Post-marketing safety monitoring and clinical risk assessment represent another critical application of RWD, enabling the detection of rare or underrecognized adverse events that cannot be identified in small-sample RCTs. The MANDALORE protocol by Convertino et al. presents a retrospective cohort analysis of SGLT2 inhibitor utilization and safety in Tuscan T2DM patients, to characterize real-world prescribing patterns, determinants of treatment switching, and adverse outcomes, which provides critical evidence for post-marketing surveillance and optimized clinical strategies. Zheng et al.‘s decade-long analysis of 59,976 vedolizumab-related reports from the FDA Adverse Event Reporting System identified 95 significant gastrointestinal AEs, 73.7% of which were not explicitly listed on the drug label. Yu et al.‘s study addressed key regulatory and clinical concerns by demonstrating that tirzepatide was associated with a 48% lower risk of suicidal ideation or attempts compared to other anti-obesity medications in a real-world cohort, providing important evidence for the psychiatric safety evaluation of this drug in clinical practice. Zufry et al.‘s five-year prospective study of insulin degludec/aspart in Indonesian T1DM and T2DM patients confirmed sustained glycemic control with a favorable safety profile, offering region-specific clinical evidence to support the rational use of this drug in Southeast Asian populations. Together, these studies collectively underscored the value of RWD in enhancing clinical vigilance and supplementing drug safety information.

**Figure 1:**
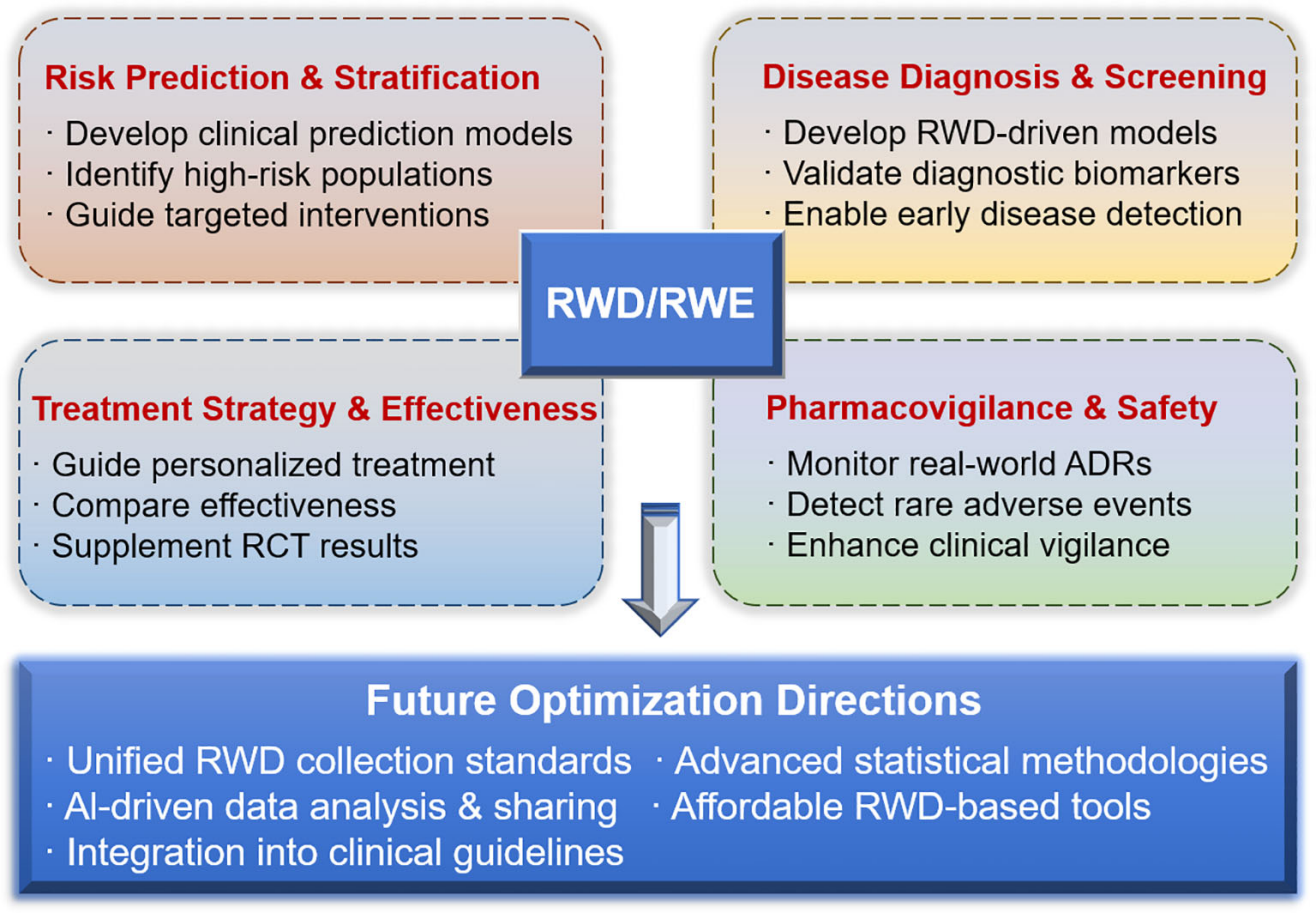
Schematic overview of the core applications and future optimization directions of RWD/RWE in endocrine disease management.

The 12 studies included in this Research Topic collectively demonstrate that RWD/RWE has evolved from a complementary research tool to an indispensable element of evidence-based endocrine medicine. While these studies illustrate the potential of RWD/RWE, their application should be interpreted with appropriate consideration of methodological constraints and context-specific limitations. By informing disease risk stratification, predictive modeling, treatment optimization, and post-marketing pharmacovigilance, RWD/RWE provides actionable insights that refine clinical decision-making and advance the integrated management of endocrine disorders. However, this will require addressing practical challenges such as acceptance by guideline developers, quality standards, and data harmonization. Further optimization of RWD/RWE is required, including establishing unified standards for endocrine RWD data collection, advancing statistical methods for confounding adjustment and causal inference, enhancing multicenter data sharing, developing affordable RWD−based tools for resource−limited settings, and integrating high-quality RWE into clinical guidelines via cross−sector collaboration (**Figure 1**). Together, these studies underscore the transformative potential of RWD/RWE in shaping evidence-based clinical decision-making and advancing the systemic management of endocrine diseases.
